# Data resulting from the CFD analysis of ten window frames according to the UNI EN ISO 10077-2

**DOI:** 10.1016/j.dib.2016.07.012

**Published:** 2016-07-16

**Authors:** Cristina Baglivo, Maria Malvoni, Paolo Maria Congedo

**Affiliations:** Department of Engineering for Innovation, University of Salento, 73100 Lecce, Italy

**Keywords:** CFD, Thermal break, Window, Frame, 10077, EPBD

## Abstract

Data are related to the numerical simulation performed in the study entitled “CFD modeling to evaluate the thermal performances of window frames in accordance with the ISO 10077” (Malvoni et al., 2016) [1].

The paper focuses on the results from a two-dimensional numerical analysis for ten frame sections suggested by the ISO 10077-2 and performed using GAMBIT 2.2 and ANSYS FLUENT 14.5 CFD code.

The dataset specifically includes information about the CFD setup and boundary conditions considered as the input values of the simulations.

The trend of the isotherms points out the different impacts on the thermal behaviour of all sections with air solid material or ideal gas into the cavities.

## **Specifications Table**

**Table:** 

Subject area	*Civil engineering,*
More specific subject area	*Dynamic analysis of frame profiles*
Type of data	*Tables, figures*
How data was acquired	*Input data are computed in according to the annex D of the UNI EN ISO 10077-2. Output data are outcomes by simulations implemented with CFD code. The computational grid is generated by GAMBIT 2.2. tool. The CFD ANSYS FLUENT Release 14.5 is used to simulate the thermal flux within the frame sections.*
Data format	*xls*
Experimental factors	*Each frame section is characterized in terms of geometry and materials.*
Experimental features	*Analysis and validation of numerical method proposed by the UNI EN ISO 10077-2 have been done.*
Data source location	*n/a*
Data accessibility	*Data is within this article.*

## **Value of the data**

• The data allow investigation of the effect of the air gas into the cavities of the frame sections.
• It is useful for a first comparison of the difference between the solid material, indicated by the UNI, and the air gas into the cavities and how impacts on the entire thermal behaviour of the frame section.
• Shows a potential method for a detailed design and evaluation of optimized frame solutions.

## 1. Data

The equivalent thermal conductivity represents the heat flow within the cavities. It is influenced by the geometry and the material. For the ten frame sections reported in the UNI, the equivalent thermal conductivity has been evaluated (File 1). CFD simulation results, in particular temperature and velocity in the cavities, are reported (File 2).

## 2 Experimental design, materials and methods

The calculation procedure based on the CFD approach has been performed by the followed steps:

1) Creation of the geometrical model.
2) Definition of the boundary conditions.
3) Numerical calculation.
4) Validation of the results.

The details of adopted methodology are presented in [1].

For each frame section, the geometry and typology (rectangular, no-rectangular or slightly ventilated) of the cavities were identified.

Thermal performance results performed by the CFD ANSYS FLUENT Release 14.5 were compared with the value provided by UNI, to demonstrate that the implemented method verifies the criteria of validation requested.

File 2 shows the isolines for the temperature, the velocity magnitude and the velocity vectors within the frame sections in two different cases: air solid and air gas into the cavities.

## Footnotes

Appendix A Supplementary data associated with this article can be found in the online version at doi:10.1016/j.dib.2016.07.012.
